# Establishing an objective decision criterion for intraocular lens exchange due to homogeneous calcification: a prospective clinical analysis

**DOI:** 10.1186/s40662-024-00415-z

**Published:** 2024-12-02

**Authors:** Timur M. Yildirim, Grzegorz Łabuz, Nikola Henningsen, Hyeck-Soo Son, Victor A. Augustin, Leoni Britz, Lizaveta Chychko, Ramin Khoramnia, Gerd U. Auffarth

**Affiliations:** David J Apple Center for Vision Research, Department of Ophthalmology, University of Heidelberg, Im Neuenheimer Feld 400, 69120 Heidelberg, Germany

**Keywords:** Cataract surgery, Contrast sensitivity, IOL complication, IOL exchange, IOL material, Patient reported outcome, Primary IOL calcification, Straylight, Visual quality

## Abstract

**Background:**

Homogeneous intraocular lens (IOL) calcification deteriorates patient’s visual quality. There is a lack of functional and patient-reported data on patients with this material change undergoing IOL exchange surgery. The aim of this study was to evaluate subjective and objective outcomes following IOL exchange due to homogeneous IOL calcification to improve evidence-based patient counseling.

**Methods:**

In this prospective, non-interventional, clinical study, 53 eyes of 42 patients with homogeneous IOL calcification were included. IOL exchange was performed in 30 out of 53 eyes. Subjective symptoms using a quality-of-life questionnaire (Catquest-9SF), photic phenomena, corrected distance visual acuity (CDVA), straylight (C-Quant, Oculus, Wezlar, Germany) and contrast sensitivity with and without glare (CSV-1000, VectorVision, Houston, USA) were assessed before (T0) and at 3 to 12 months after IOL exchange (T1).

**Results:**

Preoperative CDVA and straylight did not correlate. Average halo and glare size and intensity decreased and Catquest-9SF items improved. The CDVA rose significantly from 0.16 ± 0.13 to 0.05 ± 0.10 logMAR, and contrast sensitivity increased with and without glare. The straylight value decreased statistically and clinically significant from 2.32 ± 0.34 to 1.23 ± 0.33 log(s).

**Conclusion:**

Homogeneous IOL calcification is not always associated with a pronounced reduction in visual acuity. In most cases, IOL exchange still reduces subjective complaints and improves quality of vision of affected patients. Visual acuity should not be the sole functional parameter in assessing patients with homogeneous IOL calcification as intraocular straylight and contrast sensitivity can better objectify patients’ visual impairment. We recommend a straylight value above 1.56 log(s) as a cut-off when deciding on an IOL exchange surgery.

## Background

Intraocular lens (IOL) opacification is a significant cause of visual impairment and one of the primary reasons for IOL exchange today [1, 2]. The cause of IOL opacification differs depending on the material composition of the IOL. Microvacuole formation within the polymer is chiefly found in lenses made of low-water content, hydrophobic acrylic materials [3, 4], and calcification is associated with hydrophilic acrylic lenses [5]. According to a 2018 report, with significant geographic regional differences, hydrophilic acrylate accounts for as much as one-third of the global IOL market share [6]. The incidence of IOL calcification strongly depends on the type of lens used at a particular time and in a geographic region. It is reported to vary from 5% to 30%, reaching up to 50% in specific cohorts [7, 8]. Even when assuming a low incidence of IOL calcification, with approximately 10 million cataract surgeries undertaken each year worldwide, one can expect that perhaps hundreds of thousands of patients are affected by this condition [9]. IOL calcification was first described in the 1990s [10]. A clinical classification was proposed in 2008: primary and secondary calcification [11]. External factors cause secondary or localized calcification, while primary calcification presents as a homogenous opacity of the whole lens caused by intrinsic factors, like the IOL material itself or due to its manufacturing process. It occurs in otherwise healthy eyes several months to years after an uneventful surgery, and there are no known associated factors causing or facilitating the condition [12–15].

The only therapeutic option for affected patients is IOL exchange surgery to improve visual quality. This procedure is particularly challenging when removing a lens from the capsular bag years after implantation [16]. However, recent studies have reported that outcomes have become more predictable [17, 18]. Nowadays, similar studies report that IOL exchanges are performed more frequently with the preservation of the capsular bag.

The parameters describing an IOL’s optical performance ex vivo are the modulation transfer function (MTF) and straylight, and in vivo, the patient’s visual acuity (VA), contrast sensitivity (CS) and straylight. The impact on an IOL’s optical quality from different types of IOL calcification, has been studied ex vivo in explanted lenses, demonstrating a relatively small decrease in the MTF, the parameter clinically correlating with the VA, and a more marked effect on the straylight level [12, 19]. Assessing the VA and the patient’s subjective complaints are preferred measures to draw therapeutic conclusions in today’s clinical practice. However, despite evident morphological opacification, some patients can still reach an excellent VA of 20/20 Snellen [12]. In such cases, there is a lack of clinical data on the effects of homogenous IOL calcification on the visual function, and thus the beneficial value of IOL exchange surgery is not fully documented.

We investigated the changes in intraocular straylight in eyes with homogenous IOL calcification after IOL exchange surgery. Our study aim is to provide an aid to clinicians when deciding whether to perform IOL exchange in eyes with homogenous IOL calcification.

## Methods

### Patient enrolment

In this prospective clinical study at a tertiary eye center, patients with monocular or binocular presumed homogenous IOL calcification were included. Patients had to be older than 18 and gave written informed consent to participate in this study. Patients were excluded from the study if they had ocular comorbidities (e.g., age-related macular degeneration, diabetic retinopathy, glaucoma, or corneal dystrophy) or systemic conditions (e.g., dementia, pregnancy, or lactation) possibly impairing functional measurements. All patients were counseled on their condition and offered IOL exchange surgery. Some patients decided against surgery. The study was performed in accordance with the principles of the Declaration of Helsinki. Before commencing the study, approval was obtained from the local Ethics Committee (S-193/2022) and the collection of this patient data was registered in the Clinical Trials Register (DRKS00007837).

### Outcome measures

Patients were examined before IOL exchange surgery (T0) and 3 to 12 months after (T1). The same functional measures were performed at T0 and T1. The primary outcome measure, intraocular straylight, was assessed, averaging two consecutive measurements using an established, commercially available clinical straylight meter, the C-Quant (Oculus, Wetzlar, Germany) [20, 21]. The device has two reliability indicators for quality control: the standard deviation of the individual measuring points (Esd) and the reliability coefficient (Q). The measurement was assumed reliable when Esd was < 0.1 or Q > 0.5. If one of the indicators was out of range, the measurement was repeated. Corrected distance visual acuity (CDVA) was assessed using Early Treatment Diabetic Retinopathy Study (ETDRS) charts. CS was measured with and without glare with the CSV-1000 (VectorVision, Houston, USA). Additionally, information about the secondary IOL and intra- and postoperative complications were collected. Patient-relevant outcomes included subjective symptoms, quantification of photic phenomena using a halo and glare simulator and a quality-of-life questionnaire (Catquest-9SF).

### Surgery and handling of explants

IOL explantation with subsequent secondary IOL implantation was performed by one of two experienced anterior segment surgeons (RK and GUA) as follow: two paracenteses at 3 and 9 o’clock and a superior sclero-corneal tunnel were created. After protecting the corneal endothelium with dispersive ophthalmic viscosurgical device, the opacified IOL was moved into the anterior chamber and removed through the superior tunnel. In case of presumed vitreous prolapse, vitrectomy was performed. The secondary IOL was implanted through the tunnel and placed into the capsular bag, in the sulcus, or, in the absence of sufficient capsular support, an iris-claw-fixated IOL was enclavated horizontally behind the iris. The surgery was completed with closure of the incisions and injecting 1 mg cefuroxime intracamerally. All explanted specimens were collected, stored in balanced saline solution and transferred to the David J Apple Center for Vision Research laboratory. Calcification was confirmed following the protocols we described in previous studies [22, 23].

### Statistical analysis

The null hypothesis was that intraocular straylight at the postoperative (T1) visit remains unchanged from the value at the (T0) examination. Sample size calculation for the primary endpoint of intraocular straylight reduction was based on results from previous laboratory data. The sample size calculation indicated that, given a mean straylight level of 95.1 ± 75.6 deg^2^/sr and 5.0 ± 3.4 deg^2^/sr for calcified and clear IOLs, respectively, a total number of 22 eyes would be needed to achieve a 95% chance of detecting a difference at a 5% level of significance (two-sided *t* test) [24]. Data were handled in Excel (version 16, Microsoft, Redmond USA). Statistical analyses were performed using SPSS software (version 26; IBM Corporation). The linear regression and Pearson correlation coefficient (r) analyses of straylight and VA data were performed with MATLAB [MathWorks, Inc., USA)]. The point at which there is a 50% chance of postoperative improvement (breakeven point) of preoperative straylight and straylight improvement was determined with the Deming regression analysis using MedCalc (MedCalc Software Ltd, Belgium). Two-sided *t* tests for paired samples were used to test differences of parametric values between T0 and T1. Bonferroni corrected Wilcoxon signed-rank tests were applied for the Catquest results. A *P* value less than 0.05 indicates a statistically significant difference.

## Results

**Fig. 1:**
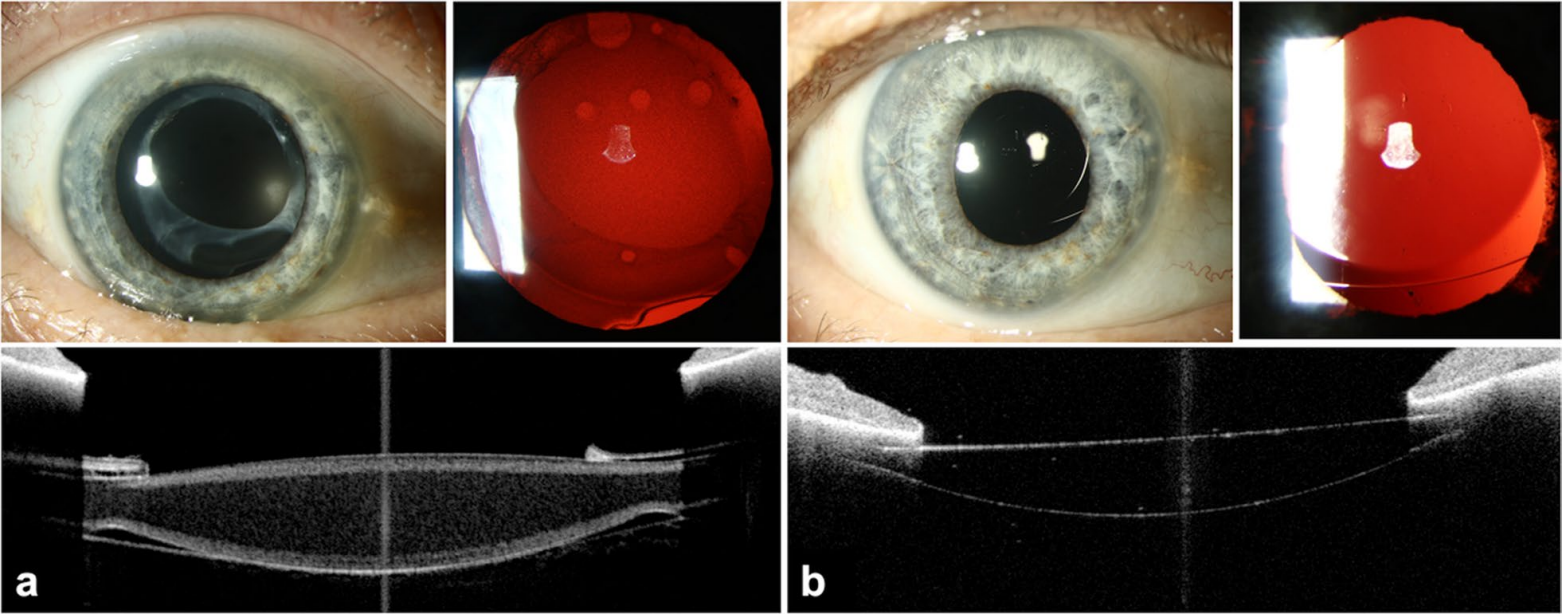
Multimodal anterior segment images. **a** Intraocular lens (IOL) with homogenous opacification (in pharmacological mydriasis). **b** Clear postoperative iris-fixated IOL (in pharmacological mydriasis); top left: slit-lamp overview photograph; top right: retroillumination photograph; bottom: optical coherence tomography cross-section image of the IOL

Fifty-three eyes of 42 patients were included in the study. All patients were pseudophakic with a hydrophilic acrylic IOL from different manufacturers. The mean age at presentation was 72.3 ± 9.1 years. Subjective complaints included cloudy or turbid vision (55%), decreased visual quality (43%), increased glare (18%), blurred vision (15%) and loss in contrast (5%). Mean CDVA was 0.11 ± 0.12 logMAR, mean straylight level was 2.30 ± 0.41 log(s). IOL exchange was performed in thirty out of 53 eyes (60%). CDVA was on average one line better in patients who did not undergo IOL exchange, while straylight values were similar between the two groups (Table 1). The choice of the secondary IOL depended on the remaining intraoperative capsular support after IOL explantation and was individually determined by the surgeon. In two cases, target refraction was − 2.5 D, the remaining eyes were planned to achieve emmetropia. In 16 cases (53%) a hydrophobic retropupillary iris-claw IOL (Artisan Aphakia, Ophtec B.V., Groningen, Netherlands) was used, hydrophobic sulcus-supported implants in 11 (37%) cases, and in 3 cases (10%) a hydrophobic capsular bag IOL was used where intact supporting tissue remained (Fig. 1). Anterior vitrectomy was performed in 27 cases (90%). Postoperative complications included: seven eyes with mild anterior chamber hemorrhage, four with small stromal iris defects, three with transient corneal edema, two with transient increase in intraocular pressure, two with hyposphagma and one with a corneal erosion. No pigment clumps or cystic macular edema were noticed in any of the eyes. In an intention-to-treat approach, all data were included in the analysis regardless of complications.

**Table 1:** Patients’ characteristics and comparison of functional parameters

Parameter	Surgery cohort *n* = 30			No surgery cohort *n* = 23	*P* ^2^
	T0	T1	*P* ^1^		
Age (years ± SD)	73.8 ± 8.3			69.9 ± 9.3	0.114*
Laterality (right: left)	20:10			11:12	0.261^#^
Gender (F : M)	58% : 42%			67% : 33%	0.581^#^
CDVA (logMAR ± SD)	0.16 ± 0.13	0.05 ± 0.10	< 0.05*	0.05 ± 0.08	0.001*
Straylight [log(s) ± SD]	2.32 ± 0.34	1.23 ± 0.33	< 0.05*	2.23 ± 0.49	0.466*

*T0* = preoperative study visit; *T1* = postoperative study visit; *CDVA* = corrected distance visual acuity; *P*^1^, T0 vs. T1; *P*^2^, surgery vs. no surgery

^*^Two-sided Student’s *t* test

^#^Fisher’s exact test.

**Fig. 2:**
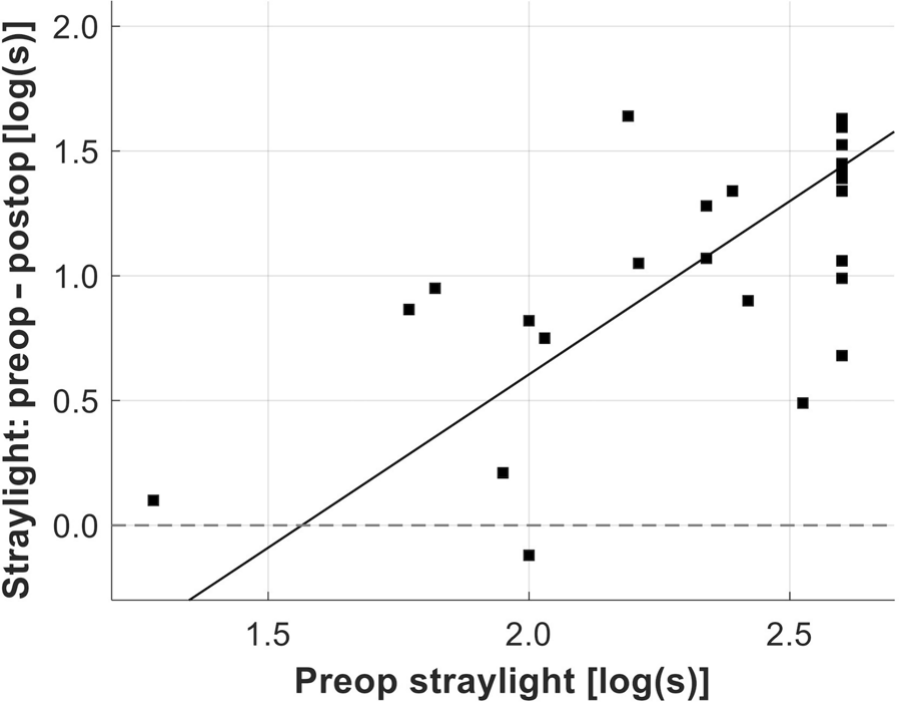
Relationship between preoperative straylight and straylight-improvement after surgery. The preoperative straylight value at which there is a 50% chance of postoperative improvement (breakeven point) was 1.56 log(s)

**Fig. 3:**
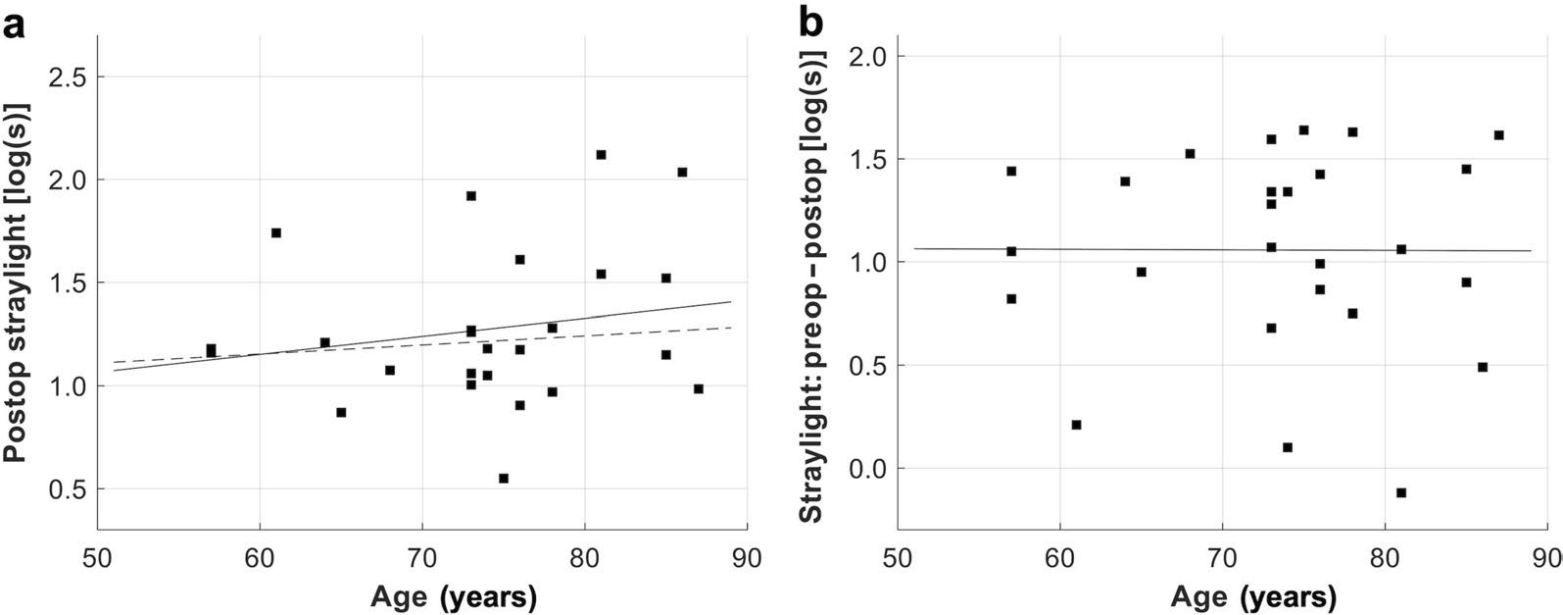
Relationship between straylight and the age of the study population. **a** The postoperative straylight values were slightly higher in older patients but close to that of healthy pseudophakic eyes (dashed line). **b** Straylight-improvement, on the other hand, was independent of age

**Fig. 4:**
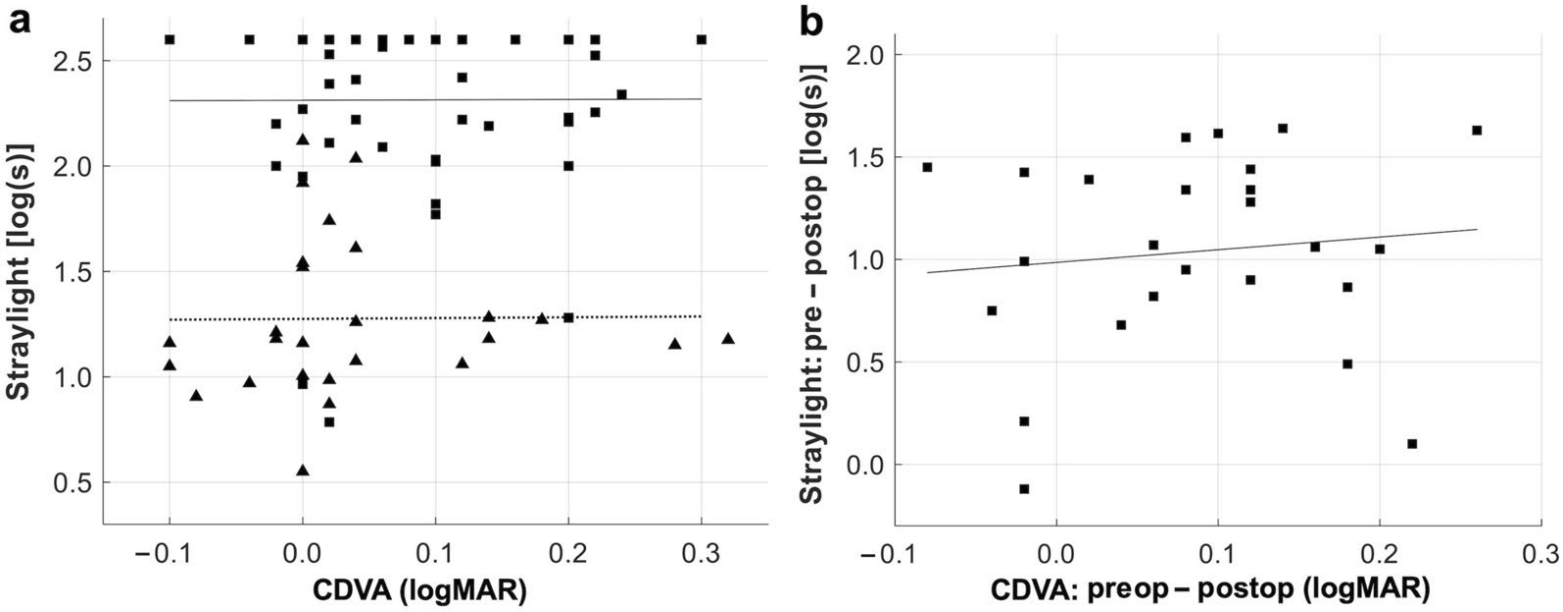
Relationship between the corrected distance visual acuity (CDVA) and straylight. **a** There is no correlation between CDVA (in logMAR) and straylight [in log(s)] indicating that both parameters are independent functional parameters. Triangles indicate preoperative, squares are postoperative values. **b** Changes in CDVA and straylight after intraocular lens exchange surgery show a slight positive correlation

**Fig. 5:**
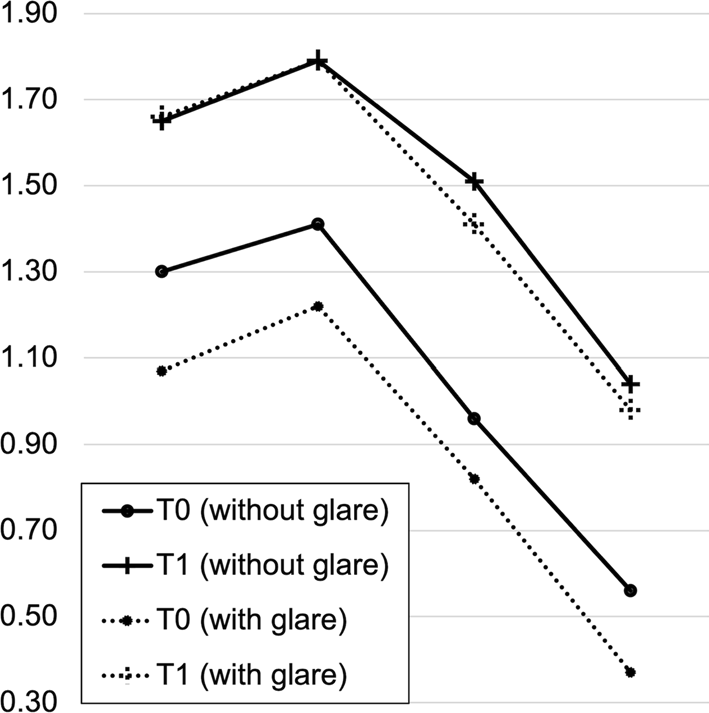
Contrast sensitivity. Contrast sensitivity with and without glare improved from T0(preoperative study visit) to T1 (postoperative study visit), with a slightly more noticeable improvement under glare conditions

**Fig. 6:**
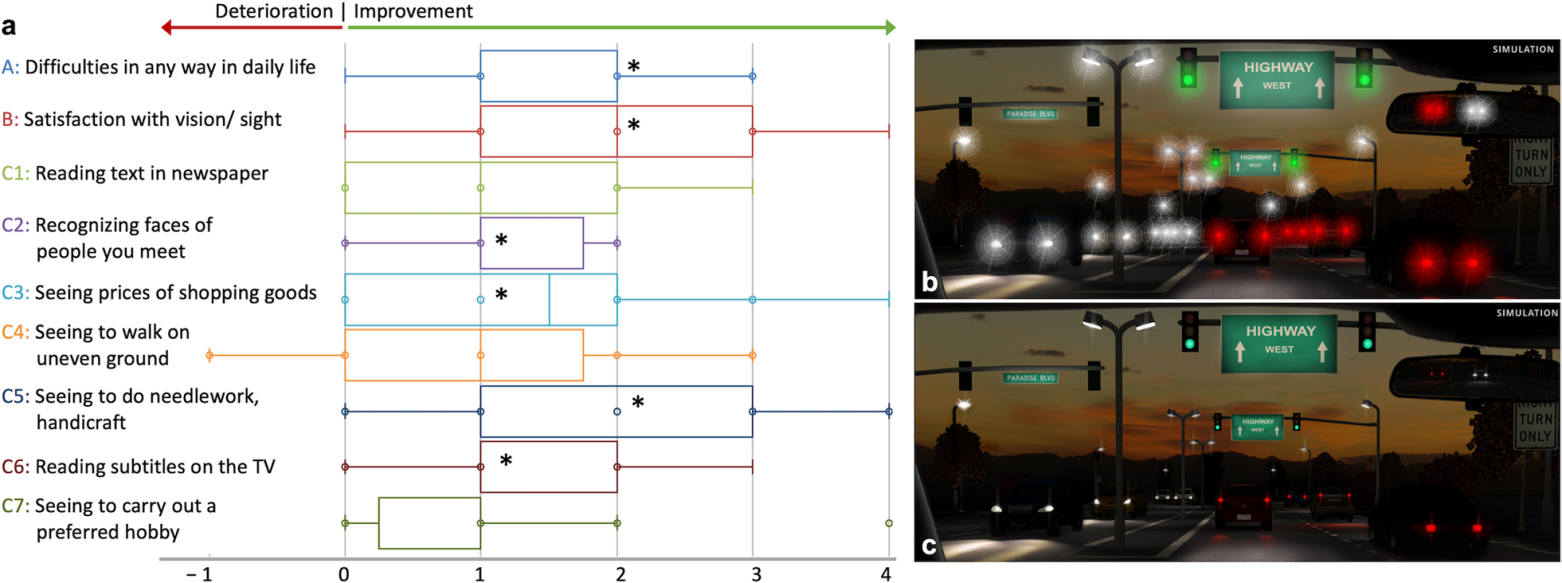
Patient-relevant outcome. **a** Catquest-9SF. Most items showed a median improvement of at least one (A, B, C2–3, C5–6); *indicates statistically significant differences between pre- and postoperative. **b** and **c** Halo and glare markedly decreased in mean intensity and size from T0 (**b**) to T1 (**c**)

Functional parameters improved in patients undergoing IOL exchange – CDVA improved from 0.16 ± 0.13 to 0.05 ± 0.10 logMAR, *P* < 0.05, as did the mean straylight value from 2.32 ± 0.34 to 1.23 ± 0.33 log(s), *P* < 0.05 (Table 1). The mean change in straylight was 1.02 log(s) (range: − 0.12 to 1.64), which did not differ significantly depending on the type of secondary IOL: the mean straylight improvement in the iris-claw group and remaining IOL types was 1.00 ± 0.46 log(s) and 1.05 ± 0.53 log(s), respectively, *P* = 0.811. A higher preoperative straylight level was correlated with a greater improvement after surgery (R^2^ = 0.30, Fig. 2). There was a tendency of a straylight increase in older patients (*r* = 0.21, Fig. 3a). Straylight improvement, on the other hand, was independent of age (*r* = 0.00, Fig. 3b). Both pre- and postoperative parameters, CDVA and straylight, did not show any correlation (*r* = 0.00 and 0.01, Fig. 4a). However, the postoperative change in both measures showed a slight positive correlation (*r* = 0.11, Fig. 4b). Mean CS with and without glare improved from the pre- to the postoperative timepoint, with a slightly more evident improvement under glare conditions (Fig. 5). CS did not differ between eyes with iris-claw and other types of secondary IOLs. The postoperative pupil size in the iris-fixated group was 3.94 ± 0.72 mm under test conditions, which did not differ significantly compared with the other IOL types (3.70 ± 0.43 mm). Postoperative spherical equivalent of eyes planned for emmetropia was − 0.05 ± 0.70 D. The average halos decreased in size and intensity, as did glare. Improvement in quality of life was seen in most Catquest-9SF items e.g., overall satisfaction with vision improved by two grades, from rather dissatisfied to very satisfied (Fig. 6). The least amount of change in straylight that was associated with subjective improvement was 0.21 log(s).

## Discussion

Currently, the decision to exchange an IOL exhibiting homogeneous calcification and the timing of such an operation are largely based on the clinician’s empirical experience. The absence of sufficient clinical data on affected eyes and their progression complicates patient counseling. In our study, patients who underwent IOL exchange showed an average improvement in mean CDVA of about one line on the ETDRS chart (from 0.16 to 0.05 logMAR). Although statistically significant, this improvement was relatively modest. Scherer et al. reported a better improvement in CVDA from 0.42 ± 0.32 to 0.25 ± 0.28 logMAR in 29 eyes that underwent IOL exchange for homogenous IOL opacification [8]. The difference in results might be attributed to our study design, which excluded eyes with additional ocular morbidities, resulting in better pre- and postoperative CDVA. Different to the CDVA, the straylight level improved significantly, both clinically and statistically, from a high mean value of 2.32 to 1.23 log(s). No prior clinical study has assessed straylight in patients with homogeneous calcification. The postoperative value in our explantation cohort is similar to a pseudophakic cohort from previous studies with a mean age of 68 years [1.21 ± 0.21 log(s)] [20]. To the best of our knowledge, there is only a single case report assessing the longitudinal straylight course of a patient with IOL calcification: in 2020, Vlasman et al. presented a case of an eye with increased straylight of 2.08 log(s) due to localized IOL calcification. The authors reduced it to 1.76 log(s) by dissolution of the posterior surface IOL deposits with a neodymium-doped yttrium aluminum garnet (Nd: YAG) laser [25]. However, in cases of homogeneous calcification, the opacity is within the IOL polymer, making Nd laser treatment inappropriate. In a previous study, we showed in eight explanted homogenously calcified segmented refractive bifocal IOLs that the opacification led to an increased mean straylight value of 2.23 log(s), which is similar to the preoperative data from this current clinical study [13]. The age dependence of straylight values in our data is in tandem with previous studies showing that older pseudophakic patients suffer from higher intraocular straylight [20]. Interestingly, the improvement in straylight was not age dependent, suggesting that functional impairment, rather than age alone, should be the primary factor in the decision to replace the IOL. Previous studies show that a straylight value of 1.5 leads to substantial visual impairment in patients’ everyday lives, especially while driving [21, 26]. A level above 1.47 log(s) was described as a serious straylight hindrance by van den Berg [21]. Patients whose ocular straylight is above that level are considered unfit to drive due to severe glare phenomena [26]. In cataract patients, previous studies suggest a preoperative breakeven point (50% chance of postoperative improvement) for straylight of 1.29 and 1.117 log(s) [27, 28]. Our data shows that patients had a 50% chance for improvement through surgery if the straylight value was above 1.56 log(s) (Fig. 2) [20]. We, therefore, recommend using this value as a reference when deciding on an IOL exchange surgery.

In addition to CDVA and straylight, testing Contrast Sensitivity adds to our understanding of the functional vision impairment in cases of homogenous IOL calcification. Unlike CDVA, which was only reduced to a small extent, CS showed a considerable reduction at all spatial frequencies, which is comparable to a nuclear opacity grade 4 cataract according to the Lens Opacities Classification System III classification system [29]. This reduction was even more pronounced under glare conditions, in accordance with the elevated straylight levels, which could explain this decrease. After IOL exchange, the CS curve with and without glare returned to a normal pseudophakic level and shape [30].

Our results show that homogeneous IOL calcification is not always associated with a distinct reduction in VA. Still, in most cases, IOL exchange reduces subjective complaints and improves the quality of vision, most likely due to a substantial decrease in intraocular straylight. CDVA and straylight are independent functional parameters, as they did not correlate. This result agrees with previous reports on opacified IOLs, suggesting that high straylight levels may indicate increased glare sensitivity in affected patients, leading to poor overall quality of vision regardless of VA [31]. Interestingly, in our study, patients who did not get an IOL exchange had a better mean CDVA than patients undergoing surgery. However, both groups had similar straylight levels, confirming that present-day decisions are mainly based on the patient’s VA. Even though the improvement of both parameters correlated to a small extent, straylight showed a more noticeable change from the pre- to the postoperative timepoint compared to VA. In their future decision-making, clinicians might focus more on the straylight value, especially in cases where the VA is not reduced, but if the patient complaints of subjective symptoms, one may consider a straylight cut-off of > 1.56 log(s) that predicts surgical success and is not dependent on the VA. The suggested threshold should be considered as an addition to facilitate the decision-making process.

Apart from these functional measures, patient-relevant outcomes are also critical in assessing the success of a procedure. In clinical studies, validated questionnaires are used for evaluating this objectively. A comprehensive review of quality assessment of ophthalmic questionnaires by Khadka et al. found that the Catquest-9SF is one of the best tools to assess patient-reported outcomes in patients with lens opacities [32]. In our study, most Catquest-9SF items improved from dissatisfied to satisfied. The overall satisfaction with vision (item B) improved by two grades. The mean improvement was similar to cataract patients before and after surgery, which corresponds with the functional measurements [33].

We believe that the data from this study will aid clinicians in judging patients’ complaints and facilitate better patient consultation.

While several case series and studies on IOL calcification exist, most deal with the localized types. A clear distinction should be made between the localized and the homogeneous type of calcification as both differ greatly in terms of patient demographics, pathogenesis, localization of the opacity, optical effects, and functional impairment [19]. Only a few retrospective clinical studies of the homogenous type: Scherer et al. provided epidemiological data like the mean interval from initial IOL implantation to the diagnosis of IOL opacification, which was 42.5 ± 19.0 months (range 1 to 84 months) and also some functional data (i.e., CDVA) before and after IOL exchange [8]. However, as the study’s main goal was to investigate potential risk factors for this condition, it did not comprehensively assess all functional and patient-relevant parameters [8]. More clinical data is needed on the course of patients undergoing IOL exchange for homogenous IOL calcification. Our study provides functional, morphological and patient-relevant outcome data, allowing a more evidence-based consultation. One strength of our study was that we only assessed the effect of the intrinsic IOL material change and the improvement through IOL exchange by excluding eyes with other ocular morbidities that could have influenced the functional measurements. However, our study has limitations: as the focus of our current study is the outcome after IOL explantation due to homogeneous hydrophilic IOL calcification, the interval from the initial IOL implantation to the time of presentation with IOL opacification was not recorded. Furthermore, the study does not allow any conclusions regarding the influence of time on the postoperative change in straylight as the patients were only examined once postoperatively. While the results we present contribute to our understanding of the subjective and objective impairment of vision that follows homogeneous IOL calcification and IOL subsequent IOL exchange, we recognize that further studies may facilitate improved treatment of affected patients even more. For example, a metric based on morphological-functional correlations could help simplify the examination process.

## Conclusion

Subjective complaints due to homogeneous IOL calcification are not always associated with reduced VA, and VA should not be the sole functional parameter used in assessing patients with IOL opacifications. Especially if surgical intervention is in doubt, patients should undergo an extended examination to determine whether IOL exchange surgery is justified. IOL exchange is usually a successful intervention for improving the quality of vision of affected patients despite good preoperative VA mainly due to decreased intraocular straylight levels after surgery. We recommend a straylight value above 1.56 log(s) as a cut-off when deciding on an IOL exchange surgery.

## Data Availability

The datasets used and/or analyzed during the current study are available from the corresponding author on reasonable request.

## Abbreviations

IOL: Intraocular lens
MTF: Modulation transfer function
VA: Visual acuity
CS: Contrast sensitivity
